# Distribution *of CCR5*-Δ*32* and HLA-B*57:01 alleles in HIV-seropositive and HIV-exposed seronegative Peruvian individuals

**DOI:** 10.1038/s41439-025-00321-3

**Published:** 2025-08-26

**Authors:** Susan M. Echavarria-Correa, Daisy Obispo-Achallma, Susan Espetia Anco, Maria Luisa Guevara, Oscar Acosta Conchucos, María Isabel Dedios, Enrique Mamani Zapana, Ricardo Fujita Alarcón, Carlos Augusto Yabar

**Affiliations:** 1 Laboratorio de Virología Clínica y Molecular, Facultad de Ciencias Biológicas, Universidad Nacional Mayor de San Marcos, Lima, Peru; 2 Laboratorio de Referencia Nacional de Virus de Transmisión Sexual, Instituto Nacional de Salud, Lima, Peru; 3 Centro de Genética y Biología Molecular, Facultad de Medicina Humana, Universidad de San Martín de Porres, Lima, Peru; 4 Hospital Santa Rosa, Lima, Peru

**Keywords:** HIV infections, Prognostic markers, Genetic markers

## Abstract

Little information is available about *CCR5-Δ32* and HLA-B*57:01 alleles in the Peruvian population, especially in human immunodeficiency virus (HIV)-negative people with high-risk sexual behavior. Here we describe the prevalence of these alleles in HIV-exposed seronegative individuals (PS) and HIV-seropositive individuals (PVV). For this purpose, 300 individuals were recruited: 150 from each group, and the selected alleles were characterized by endpoint PCR, real-time PCR and DNA sequencing. According to our results, the prevalence of *CCR5/CCR5-Δ32* heterozygous was 2.7%, and no homozygous cases were found. The population was in Hardy–Weinberg equilibrium for the *CCR5* locus. Regarding HLA-B*57:01, one case was identified in the PS group, while no cases were observed in the PVV group. No statistical difference was detected between groups (*P* > 0.05). In conclusion, we showed a low prevalence for *CCR5-Δ32* as HLA-B*57:01 in the Peruvian population. As these alleles were found at similar frequencies among both HIV-positive and HIV-negative Peruvian individuals with high-risk sexual behavior, it is possible that other genetic factors play an important role in preventing HIV transmission in this population. The low frequency of the HLA-B*57:01 allele in the Peruvian population suggests that routine genotyping tests for abacavir hypersensitivity should be reevaluated in the public health policies of Peru’s Ministry of Health, based on national epidemiological data.

## Low prevalence of CCR5-Δ32 and HLA-B*57:01 in Peru

Some people are naturally resistant to human immunodeficiency virus (HIV). This resistance might be due to certain genes, such as *CCR5 Δ32* and HLA-B*57:01. The *CCR5 Δ32* gene can prevent the virus from entering cells, while HLA-B*57:01 is linked to slower disease progression and reactions to a drug called abacavir. In Peru, the government requires testing for HLA-B*57:01 before prescribing abacavir, but the prevalence of this gene in Peruvians was unknown. Researchers conducted a study with 300 Peruvian participants, including both HIV-positive and high-risk HIV-negative individuals. They used genetic tests to check for the presence of *CCR5 Δ32* and HLA-B*57:01 and found that both genes are rare in Peruvians, with *CCR5 Δ32* present in 2.7% of participants and HLA-B*57:01 in only 0.33%. The study suggests that these genetic traits are uncommon in Peru owing to limited European ancestry.

This summary was initially drafted using artificial intelligence, then revised and fact-checked by the author.

## Introduction

Genetic factors have been implicated in resistance to human immunodeficiency virus (HIV) infection despite multiple high-risk exposures, as well as in differing rates of progression to acquired immune deficiency syndrome (AIDS) among infected individuals^1^. Genes such as *CCR5 Δ32* and HLA-B*57:01 have been implicated in viral entry and immune response processes, respectively^2^. In the first case, a 32-bp deletion in the CCR5 gene is sufficient to interfere with, and in some cases prevent, viral entry into the cell—particularly in individuals who are homozygous for the mutation^1^. The HLA-B*57:01 allele is associated not only with slow progression to AIDS^3^ but also with genetic susceptibility to the antiretroviral drug abacavir (ABC) hypersensitivity reaction (ABC-HSR)^4^, which is known to cause severe clinical manifestations^5^. For this reason, HLA-B*57:01 genotyping is recommended before prescribing the drug^6^. In Peru, the Ministry of Health has developed the technical standard NTS N°169-MINSA/2020/DGIESP^7^, which establishes the requirement to perform the HLA-B*57:01 genotyping test before administering ABC. However, this political decision was made without having proven that HLA-B*57:01 frequency in the Peruvian population.

In contrast to Europe, the prevalence of both *CCR5*-Δ*32* and HLA-B*57:01 is low in African, Asian and Latin American populations^8^. In Peru, this mutation may have been introduced into the local population by gene flow through admixture with the European population^9,10^. This is supported by few reports found in some Peruvian individuals^8,11^. However, none of these studies included the geographic origin of the recruited participants, which complicates interpretation, as the Peruvian population comprises communities with varying degrees of ethnic admixture and, consequently, genetic variability^12^. Moreover, in the context of HIV/AIDS disease, none of these studies included clinical or socioepidemiological data that could help clarify the relevance of these alleles in the evolution of the epidemic in Peru.

For this study, we recruited Peruvian individuals and collected data on their geographic origin as well as social, epidemiological, and clinical characteristics to better understand how the genetic makeup of the Peruvian population relates to the HIV/AIDS epidemic

## Materials and methods

### Type and design of study

This observational cross-sectional study was performed between December 2020 and November 2021.

### Population and sample size

The study population consisted of Peruvian individuals treated at the *Asociación Civil Voluntades Lima Norte* Non-Governmental Organization and the *Santa Rosa* Hospital. This population was divided into two groups: (1) HIV-exposed seronegative individuals (PS) and (2) HIV-seropositive individuals (PVV). The geographic origin of the participants was predominantly Lima (71%), a city characterized by a highly admixed population and the greatest genetic diversity in the country^12^. From this population, the sample size was calculated using the proportion formula for finite population based on (1) an expected prevalence of 1% for HLA-B*57:01 and *CCR5*-Δ*32* in a mixed population (mainly with native ancestry), (2) a population of 800 individuals, (3) a confidence level of 95% and (4) an error margin of 1%. The minimum number of participants required to estimate the prevalence of *CCR5*-Δ*32* and *HLAB-57:01* alleles was 258; however, in this study a sample size of 300 individuals was used to increase the power and precision of the results. The selection criteria were the following: (1) Peruvian nationality, (2) legal age (18 years or more), (3) both sexes (male and females) and (4) agreement to participate in the study by signing an informed consent and completing a survey using a data collection instrument. Furthermore, for the PVV group, HIV seropositivity was confirmed through diagnostic tests, whereas for the PS group, seronegativity was confirmed by both third- and fourth-generation rapid tests, along with documented high-risk sexual behaviors such as occasional contact with sex workers, intermittent condom use and multiple sexual partners.

### CCR5 genotyping

DNA was extracted using the NucleoSpin kit (Macherey-Nagel), according to the manufacturer’s recommendations. *CCR5* genotyping was based on endpoint polymerase chain reaction (PCR) assay using primers flanking the 32-bp deletion, CCR5 DELTA1 (5′-ACCAGATCTCTCAAAAAGAAGGTCT-3′) and CCR5 DELTA2 (5′-CATGATGGTGAAGATAAGCCTCCACA-3′)^2^. The reaction included 0.2 µM of each primer, 0.04 U of Velocity DNA polymerase enzyme, 2.5 mM Mg^2+^ and 0.6 mM dNTP mixture in a final volume of 25 µl. The cycling parameters were: 1 cycle 98 °C × 30 s; 35 cycles 98 °C × 30 s, 60 °C × 30 s, 72 °C × 15 s; 1 cycle of 72 °C × 3 min. The amplified products were visualized by 3% agarose gel electrophoresis. For the *CCR5*/*CCR5* genotype, a single band of 225 bp is expected; for the *CCR5*/Δ*32* genotype, bands of 225 bp and 193 bp; and for the *CCR5*-Δ*32/ CCR5*-Δ*32* genotype, a single band of 193 bp.

### HLA-B*57:01 allele genotyping

HLA-B*57:01 genotyping was performed using a modified procedure based on Jung et al.^13^. The PCR reaction included 10–60 ng/μl of genomic DNA, 1× Kapa Probe Fast Master Mix (Roche), 0.3 μmol/l of each primer (B5071-T1F: AGGGTCTCACATCATCCAGGT and B5701-T3R: CGTTCAGGGCGATGTAATCCT) and 0.2 μmol/l of each probe (B5701-P2: 6FAM-CGCGGGCATGACCAGTC-MGBNFQ), with a final volume of 10 μl. The amplification program had the following parameters: 95 °C for 5 min; and 45 cycles of 95 °C for 15 s and 68 °C for 30 s. Molecular-grade water was used as a negative control, and a previously genotyped HLA-B*57:01 (+) sample (confirmed by a commercial kit) served as the positive control. Samples with a Ct value <30 for both HLA-B*57:01 and the internal control (alpha-actine 1) were considered as positive for the HLA-B*57:01 allele.

### Sanger sequencing

DNA samples whose PCR product revealed the presence of heterozygous genotype for *CCR5*-Δ*32* were purified from gel. The primers described by Diaz et al. were used^2^. In the case of HLA-B*57:01, the amplification products were directly purified using magnetic beads and primers described by Jung et al.^13^. In both cases, Big Dye Terminator reagent was used, and the products were settled on the Applied Biosystems 3500 XL genetic analyzer. In the case of *CCR5*, both forward and reverse sequences were edited and assembled with the software SeqTrace v.0.9.0, whereas, for *HLA-57:01*, we used the free-access program SoapTyping V1.0.6.3. The resulting electropherograms were aligned and compared with the sequence deposited in GenBank (accession code LR961919, information detailed in the link:https://www.ncbi.nlm.nih.gov/nuccore/LR961919).

### Statistical analysis

Comparison of categorical and quantitative variables was performed with the chi-square test or Fisher’s test (*f* < 5), and the Mann–Whitney test, respectively. Deviations from Hardy–Weinberg equilibrium were calculated using the chi-square test. Frequencies of each allele were compared between HIV-exposed seronegative and HIV-seropositive individuals, using chi-square test or Fisher’s test when appropriate. In all analyses, *P* values <0.05 were considered statistically significant. Finally, a new sample statistical power calculation was performed using the actual allele frequencies observed. For this, the power of a two-sided Fisher’s exact test (exact2x2) was calculated using RStudio v4.1.2. The calculation assumed that *CCR5Δ32* confers resistance, with the alternative hypothesis set to a one-sided test within the same function call.

## Results

The data of the 300 participants are summarized in Table 1. For the PVV groups, the mean age was 36 years (83.3% males and 16.7% females), while for the PS group, the mean age was 33 years (84.7% males and 15.3% females). Most of the participants in either the PVV or the PS came from Lima (64%), followed by Lambayeque (6%) for the PVV, Ica for the PVV and PS (4% each) and La Libertad and Loreto for the PVV and PS (3% each). Regarding risk behavior, no information was available for the PVV group; however, in the PS group, 36% reported homosexual orientation (men who have sex with men (MSM) and Trans H-M), 10.7% bisexual, and 53.3% heterosexual. Both groups showed statistically significant differences in chronological age (*P* = 0.02) and immunological status (*P* < 0.01), indicating immunological failure in PVV.

**Table 1:** Sociodemographic, epidemiological, clinical and virological characteristics of PVV and PS groups.

Characteristics	PVV (*n* = 150)	PS (*n* = 150)	*P*^a^
**Gender**			
**Male**	83.3%	84.7%	n.s.^b^
**Female**	16.7%	15.3%	
**Age (average) [IQR]**	36 [29–45]	33 [26–41]	0.02^c^
**Sexual orientation**			
**MSM**	No data	20.7%	
**Trans H-M**	No data	15.3%	
**Bisexual**	No data	10.7%	
**Heterosexual**	No data	53.3%	
**Viral load (number of copies/ml)**			
**≤50**	54%	Not applicable	
**>50**	13.3%	Not applicable	
**Unknown**	32.7%	Not applicable	
**CD4 recount (cell/µl) (average) [IQR]**	618 [378.5–785.25]	862 [706–1,086]	<0.01^c^
**<200**	8%	0%	
**200–500**	25.3%	8%	
**>500**	66%	92%	
**Unknown**	0.67%	0%	
**Place of birth**			
**Amazonas**	0.6%	0	
**Ancash**	4.7%	2%	
**Apurímac**	0.6%	1.3%	
**Arequipa**	2.0%	0.6%	
**Cajamarca**	2.7%	0	
**Callao**	2%	2%	
**Huánuco**	2.7%	0.6%	
**Ica**	2%	4%	
**Junín**	1.3%	2.7%	
**La Libertad**	3.3%	0.6%	
**Lambayeque**	6%	1.3%	
**Lima**	64%	76.7%	
**Loreto**	2.7%	3.3%	
**Pasco**	0	0.6%	
**Piura**	2%	2%	
**San Martin**	1.3%	0.6%	
**Tumbes**	1.3%	0%	
**Ucayali**	0.6%	1.3%	
**ABC treatment**			
**Si**	6	Not applicable	
**No**	144	Not applicable	

^a^*P* value using chi-square. ^b^Chi-square test. ^c^Mann–Whitney *U* test (*P* < 0.05 denotes statistically significant difference). n.s., no statistically significant difference.

**Fig. 1:**
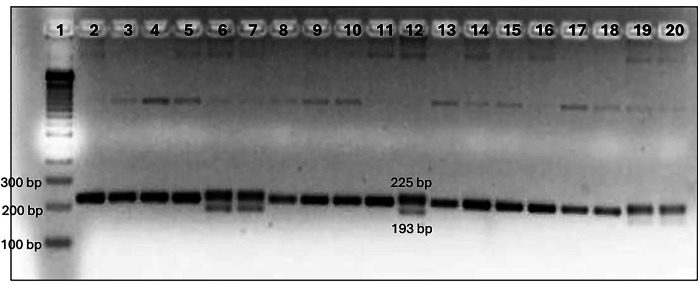
*CCR5* genotype. Lane 1: 100-bp ladder. Lanes 2–11 and 13–20: homozygous genotype wild-type *CCR5/CCR5*. Lanes 6, 7 and 12: heterozygous genotype mutant (*CCR5/CCR5-Δ32*).

**Fig. 2:**
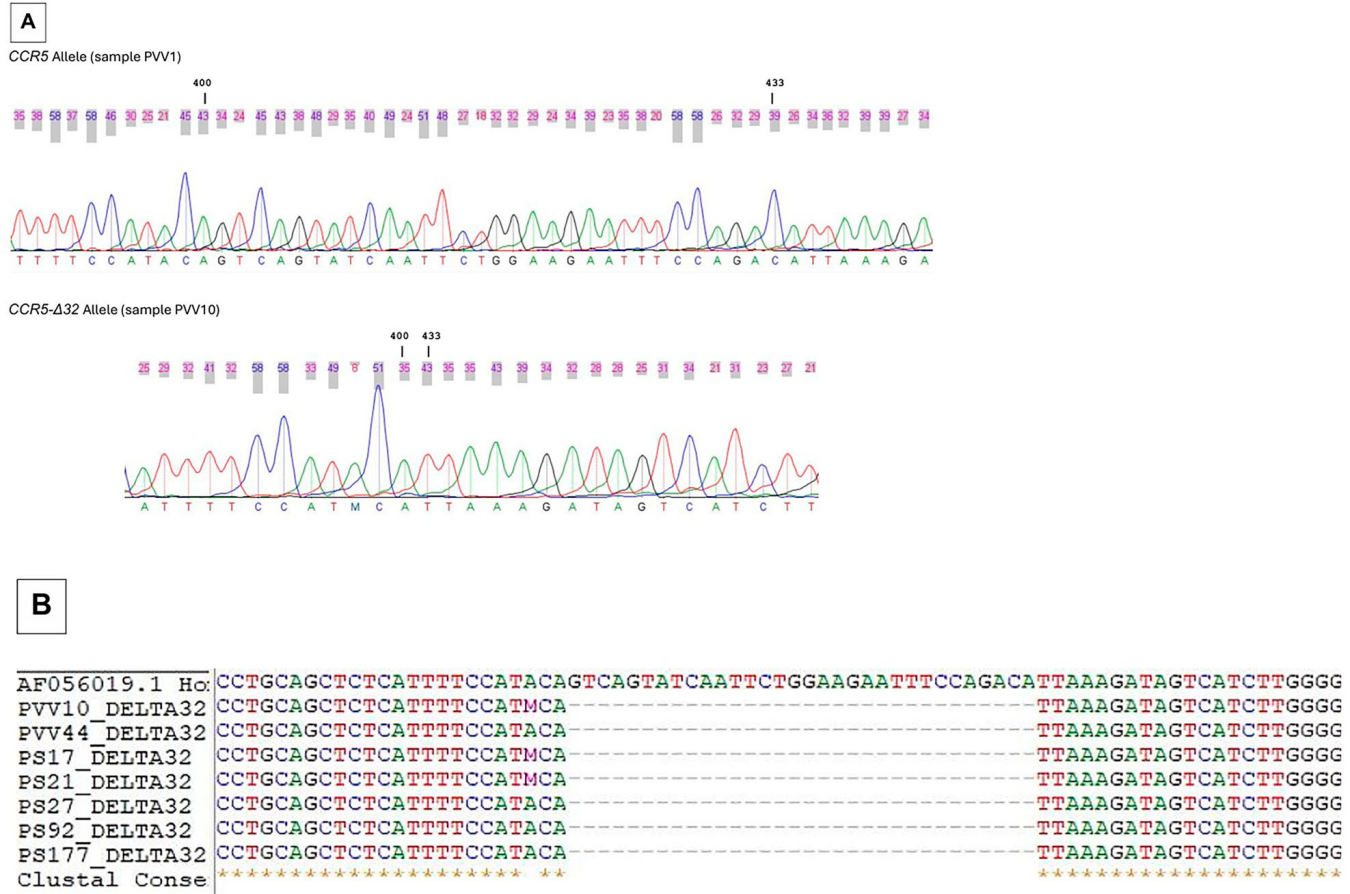
Sanger sequencing of the *CCR5*-Δ*32* allele*.* **a**, Electropherograms corresponding to *CCR5* allele and *CCR5*-Δ*32* allele visualized by SeqTrace software. **b**, Alignments of *CCR5*-Δ*32* using Bioedit software.

The *CCR5*/*CCR5* genotype was observed as a single band of 225 bp, whereas the *CCR5*/*CCR5*-Δ*32* genotype was visualized as two bands (225 and 193 bp) (Fig. 1). No *CCR5*-Δ*32*/*CCR5*-Δ*32* genotype were detected in the 300 samples. The mutated allele (*CCR5-Δ32*) was further confirmed by Sanger sequencing (Fig. 2).

We found that the prevalence of *CCR5 Δ32* heterozygotes was 2.7%, while the frequency of the allele was 1.3% (Table 2). No homozygotes were found for the mutated allele. In the PVV group, 2% had the heterozygous genotype and the frequency of the *CCR5-Δ32* allele was 1%. In the PS group, 3.3% had the heterozygous genotype and the frequency of the *CCR5-Δ32* allele was 1.7%. The distribution of genotype and allele frequencies between the two groups was not statistically significantly different (*P* > 0.05). To determine if the lack of statistical significance was due to insufficient statistical power, we recalculated the statistical power of Fisher’s exact test as described in the ‘Materials and methods’ section. Considering a one-sided test (alternative = ‘one.sided’), the power was determined to be 0.091, requiring a total of 3304 per group to achieve a statistical power of 80%.

**Table 2:** Allele and genotype frequencies of *CCR5* between PVV and PS and Hardy–Weinberg equilibrium (HW).

Group	*n*	Genotypic frequencies				Allelic frequencies			HW equilibrium	
		CCR5/CCR5	*CCR5*/*CCR5*-Δ*32*	*CCR5*-Δ*32*/*CCR5*-Δ*32*	*P*^a^	*CCR5*	*CCR5*-Δ*32*	*P*^a^	*χ*^2^	*P*^b^
**PS**	150	145 (0.967)	5 (0.033)	0	n.s.	0.983	0.017	n.s.	0.043	n.s.
**PVV**	150	147 (0.98)	3 (0.02)	0		0.99	0.01		0.015	n.s.
**Total**	300	292 (0.973)	8 (0.027)	0 (0)		0.987	0.013		0.055	n.s.

^a^*P* value using Fisher’s test. ^b^*P* value obtained by chi-square. n.s., nonsignificant statistical difference.

Meanwhile, Hardy–Weinberg analysis showed that the *CCR5-Δ32* allele was in equilibrium within each group and across the total population (>0.05) (Table 2).

**Fig. 3:**
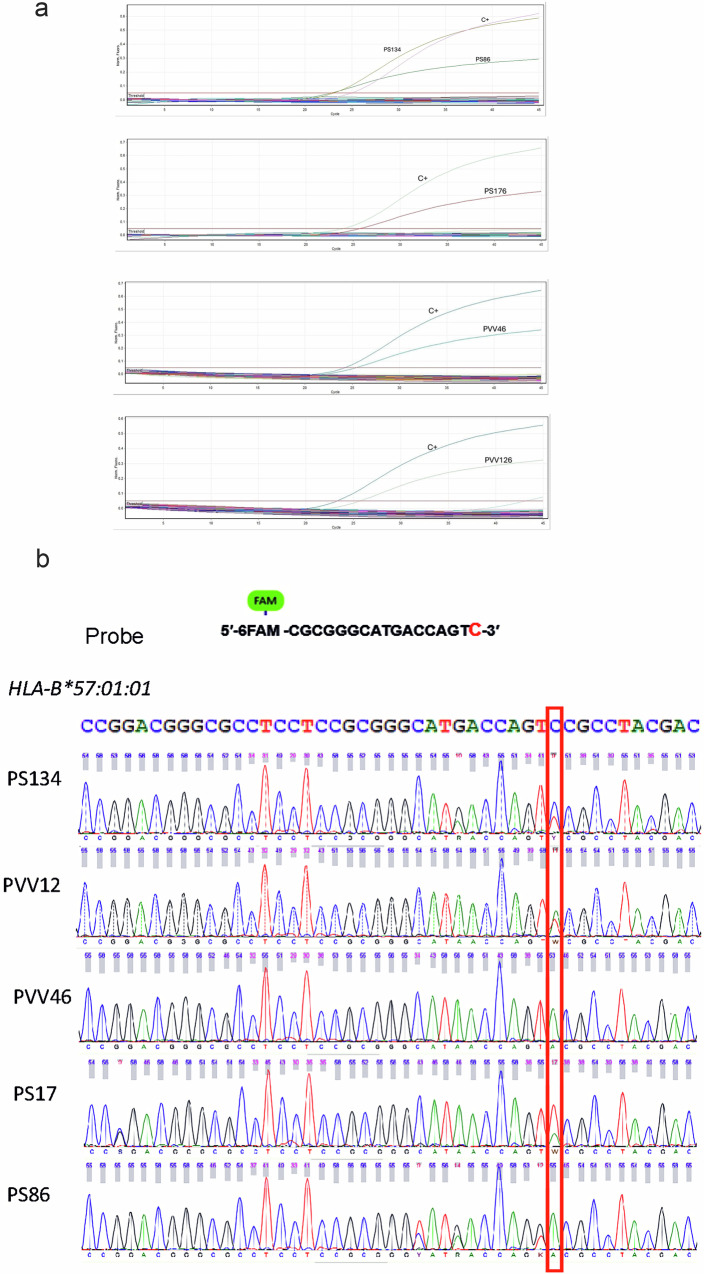
Genotyping of HLA-B*57:01. **a** Real-time PCR results. The sample PS134 is the only one with a similar fluorescence level as the positive control (C+). **b** Comparison of nucleotides according to electropherogram peaks with the HLA-B*57:01:01 sequence extracted from GenBank. The red rectangle indicates the position of the SNP (C/T) for HLA-B*57:01:01.

Real-Time PCR results showed Ct values below 30 for both the *HLA-B57:01* allele and the internal control in 5 of the 300 analyzed samples. However, only one of these samples (PS134) showed a fluorescence level comparable to the positive control (Fig. 3a); hence, Sanger sequencing was used to avoid false positives.

Analysis using the SoapTyping program revealed matches to the *HLA-B57:01* allele in only one of the five samples (data not shown). Based on the electropherograms, we confirmed that this sample had polymorphisms compatible with the sequence of the HLA-B*57:01:01 allele stored in the GenBank platform (accession number LR961919), whereas the remaining four samples had noncompatible polymorphisms in the region complementary to the 3′ end of the probe (Fig. 3b). Therefore, we consider that sample PS134 carries the HLA-B*57:01 (+) genotype.

Overall, the prevalence of the HLA-B*57:01 genotype was 0.33%. In the PS group, the prevalence was 0.67%. No HLA-B*57:01 genotype was identified among HIV-seropositive individuals (PVV). The prevalence between the two groups was not statistically significantly different (*P* > 0.05) (Table 3). Similarly to *CCR5-Δ32*, we performed a new calculation of the statistical power of the sample based on the identified HLA-B*57:01 frequency value. According to our results, the statistical power was 0.003507617, indicating that a sample size greater than 50,000 would be required.

**Table 3:** Prevalence of HLA-B*57:01 in HIV-seropositive individuals (PVV) and HIV-exposed seronegative individuals (PS).

Group	*N*	Genotype HLA-B*57:01		Prevalence (%)	*P*^a^
		Positive	Negative		
PVV	150	0	150	0	n.s.
PS	150	1	149	0.67	
Total	300	1	299	0.33	

^a^*P* value with Fisher’s test. n.s., not statistically significant.

## Discussion

In the present study, we have described the *CCR5*-Δ*32* and HLA-B*57:01 alleles in HIV-exposed seronegative and HIV-1 seropositive Peruvian individuals and collected social, clinical and epidemiological information for each participant. In the case of *CCR5*-Δ*32*, our findings reveal that the prevalence of this mutation was low, which could be related to the low degree of admixture detected between the current Peruvian population and the European population, primarily Spanish^10^. The sample of Peruvian individuals analyzed in this study did not present any homozygous case for the CCR5 mutation, consistent with the results from previous studies in Latin American countries^8,14,15^.

In contrast to our data, Solloch et al.^8^ reported a higher prevalence for *CCR5-Δ32* (5% versus 3%). This difference, albeit small, could be related to the methodology used, the size of the sample and the selection of the population. In addition, Solloch et al.^8^ did not provide further information on the individuals’ backgrounds, making it difficult to assess how the degree of admixture among Peruvians varies by geographical origin. In our study, we included participants from 18 cities in Peru, primarily from Lima, Lambayeque and Loreto—regions known for having the highest genetic variability in the country^12^. These data suggest that the selected populations might be representative of Peru; however, further nationwide studies are needed to confirm this.

Regarding the HLA-B*5701 allele, our study showed a lower frequency than *CCR5-Δ32*, which could be due to its higher distribution in Caucasian populations than in Latin American or Native American populations^16,17^. The allele frequency in some Latin American countries ranged from 1% to 5.6% (refs.^16–19^), suggesting that the degree of admixtures with the European lineage is variable. Martínez et al.^17^, who reported a prevalence of 2.7% among HIV-infected Colombians, found that, when the sample was stratified according to ethnic characteristics, the prevalence was higher in white individuals (4%) than in other ethnic groups such as mestizos (2.6%) or Afro-Colombians (1.9%). These results show that the allele distribution in Latin America may differ depending on the local ethnic characteristics of each country, suggesting that the degree of admixture in this population is higher than in other regions of the world.

On this last point, the study published by Vilcarino et al.^11^ found a frequency of 4% in a sample of 49 Peruvian participants. However, the data on the geographical origin of these samples are limited, as most of them included only individuals from the same city. In addition, the sample size was very small, so this value could not be considered representative of the Peruvian population. By contrast, the sample size in this study was larger (*n* = 300) and from different regions of Peru (Table 1). Considering that the Allele Frequency Net database (https://www.allelefrequencies.net/) lacks data on the prevalence of this allele in Peru, this study may be the first to provide approximate prevalence estimates of HLA-B*57:01 in the Peruvian population.

From a clinical point of view, the presence of both *CCR5*-Δ*32* and HLA-B*57:01 have been associated with slow progression to AIDS in carrier individuals^1,3^. Their presence in the Peruvian population opens the possibilities of finding a specific group of individuals with slow progression to AIDS. However, among carriers of the *CCR5-Δ32* mutation, only one exhibited a viral load below 20 copies, while the others had viral loads exceeding 1000 copies. It is therefore possible that pharmacological and/or genetic factors may influence virological failure in carriers of this mutation.

The CD4 count was statistically significantly lower in PVV, which is to be expected because HIV infection is associated with a depletion of the CD4 lymphocyte population^20^. However, when analyzing the immune response status of *CCR5*-Δ*32* mutation carriers in the PVV group (PVV^*CCR5/Δ32*^, *n* = 3), they showed more than 400 CD4^+^ cells/µl, a value outside the threshold for risk of developing AIDS^21^, and above the average CD4 of those who did not carry this mutation. The high proportion of CD4^+^ cells may be a consequence of CD30L overexpression associated with the 32-nucleotide deletion^22^. Another important detail related to the immune response is that the antiretroviral treatment currently being received by PVV^*CCR5/Δ32*^ based on a combination of dolutegravir, tenofovir, lamivudine and efavirenz may also contribute to favoring the immune response against HIV, as has been described in a previous study^23^. However, ex vivo experiments have shown that treatment with dolutegravir has the opposite effect, generating a decrease in the CD4 population^24^. Considering that two individuals in the PVV^*CCR5/Δ32*^ group were treated with this drug and still maintained a CD4 count greater than 400 cells/µl, we suggest that the CD4 population restoration is attributable to the mutation.

We collected the data regarding the risk behavior of *CCR5-Δ32* mutation carriers from the PS group (PS^*CCR5/Δ32*^, *n* = 5). They reported high-risk sexual behavior for HIV/Sexual Transmitted Infections such as multiple sexual partners, infection by sexual transmission, use of recreational drugs and intermittent condom use. In spite of this, they had a good immune response (an average of 1,011.4 cells/µl) and a negative diagnosis of HIV at quarterly or half-yearly monitoring tests. These characteristics may be associated with the *CCR5-Δ32* mutation, which, according to this study, was more prevalent in PS than in PVV, although not statistically significantly so. Because the statistical power calculation yielded a value of 0.091, this finding suggests a possible false negative due to the small sample size (*n* = 300) in this study. However, the possibility of other genetic or immunological factors contributing to a degree of resistance to PS should not be discounted. In fact, a recent exome study revealed a new allele HLA-DQB1*03:419 found in the Peruvian population associated with immunological protection (*P* < 0.04)^25^. These findings highlight the need for further genomic research to identify new protective genes in different geographic regions of Peru.

The presence of a single individual with the HLA-B*57:01 allele among 300 HIV-positive and HIV-negative Peruvian individuals (0.3%) indicates the rarity of this allele in the Peruvian population. This fact may be related to the population’s predominantly indigenous genetic characteristics, even among the mestizo population, as demonstrated in previous studies^12,26^. Considering the approximate HIV prevalence in the Peruvian population is 0.3% and that 89% of those diagnosed have access to antiretroviral therapy^27^, the administration of ABC in this group would be exceptional. In fact, only 6 of the 150 HIV-positive participants in our study received ABC, confirming the limited access to this drug. Moreover, the Peruvian Technical Normative on Antiretroviral Therapy Recommendations does not mandate the use of this drug in first- or second-line therapy, which could explain why Peruvian doctors prescribe it as a second alternative, among other clinical reasons.

Regarding the administration of ABC, cohorts including more than 200,000 participants, mostly Caucasian, revealed that only 5–8% of cases presented hypersensitivity symptoms when treated with ABC^28^. Likewise, only 6% of those with the mutation who manifested hypersensitivity reactions are at high risk^29^.

Regarding the financial sustainability of the HLA-B*5701 test, it has been shown to be cost-effective in a US population^30^; however, these estimates could vary in populations with high levels of interbreeding, such as the Peruvian population. In fact, a significant proportion of the Peruvian population is of indigenous descent, even among the mestizo population^12^. Together, these data suggest reconsidering the value of routinely detecting this allele within Peru’s resistance surveillance and monitoring system, unless multicenter studies with representative sample sizes are conducted to define its national distribution. Given that our results indicate that achieving 80% statistical power requires recruiting over 50,000 participants, the cost-effectiveness of HLA-B*5701 testing for routine use could be disregarded. Based on the evidence from this study and current ABC management practices in Peru, it is possible that hypersensitivity cases in this country are very rare. However, these findings should be viewed with caution.

Finally, this study focuses on the genetic variants *CCR5-Δ32* and HLA-B*57:01 in PVV and PS groups, and the results presented will support future studies in estimating more representative sample sizes across different geographical regions of Peru. It also demonstrates the need for local studies in search of markers to better understand HIV resistance in at-risk populations and to predict the prognosis of AIDS in the Peruvian population. The effects of *CCR5*-Δ*32* in Peruvian individuals may differ from those described in European populations, due to genetic heterogeneity. These effects are influenced not only by allele frequency but also by interactions with other genes that may modulate *CCR5*-Δ*32* expression individually or synergistically^31^.

In conclusion, our findings show that the prevalence of both *CCR5-Δ32* and HLA-B*57:01 alleles is low, supporting evidence of limited recent gene flow between European populations and indigenous communities in present-day Peru. Similarly, the presence of one carrier of the HLA-B*57:01 allele—and two previously reported cases—demonstrates the ongoing surveillance of ABC hypersensitivity in the Peruvian population. However, further nationwide investigations covering each region of Peru are needed to obtain a truly representative sample of the Peruvian population.
